# Approaching to biogenic amines as quality markers in packaged chicken meat

**DOI:** 10.3389/fnut.2022.966790

**Published:** 2022-09-01

**Authors:** Luigi Esposito, Dino Mastrocola, Maria Martuscelli

**Affiliations:** Faculty of Bioscience and Technology for Food, Agriculture and Environment, University of Teramo, Teramo, Italy

**Keywords:** biogenic amines, chicken meat, quality, safety, BAI index, TBARS, packaged meat, refrigerated meat

## Abstract

Following the chicken meat quality decay remains a tricky procedure. On one hand, food companies need of fast and affordable methods to keep constant higher sensory and safety standards, on the other hand, food scientists and operators find difficult conjugating these exigencies by means of univocal parameters. Food quality definition itself is, in fact, a multi-layered and composite concept in which many features play a part. Thus, here we propose an index that relies on biogenic amines (BAs) evolution. These compounds may indirectly inform about microbial contamination and wrong management, production, and storage conditions of meat and meat products. In this study, three cuts of chicken meat (breast filets, drumsticks, and legs) packed under modified atmosphere, under vacuum, and in air-packaging, stored at +4°C (until to 15 days), were analyzed. Some BAs were combined in an index (BAI) and their evolution was followed. The Thiobarbituric Acid Reactive Species assay (TBARS) was also used as a common reference method. Generally, BAI may better identify the beginning of quality impairment than lipid oxidation spreading. ANOVA statistical analysis has highlighted that the storage time is anyway the most detrimental factor for chicken decay when it is stored in refrigerated rooms (*p* > 0.01). Despite TBARS still remains a powerful tool for chicken goods, its exclusive use may not be enough to explain quality loss. On the contrary, BAI implementation in fresh meat can give a more complete information combining food safety exigencies with sensory attributes.

## Introduction

By looking at data from FAO-OECD, chicken meat represents a vital food commodity equally distributed in all countries. Its popularity is conditioning the meat market trends covering the 40% of total protein demand globally (1). Chicken meat ensures a balanced nourishment with a limited caloric intake and a reduced price (2). Moreover, it respects all religious requirements (3), its production is easier, and besides the forecasted production increase, this sector is the one on which more effective policies can be actuated for climate change mitigation (4). Despite its success, from the quality point of view (5), chicken meat marketing suffers of problems related to stability and sensory impairment. Chicken meat is normally more susceptible of microbiological spoilage (6), moreover, muscular tissues are characterized mainly by monounsaturated (MUFAs) and polyunsaturated fatty acids (PUFAs) which are rapidly oxidated by O_2_ and light (7, 8). Thus, the development to rancidity is a real problem for the poultry meat industry interesting both transformed products and fresh meat (9). In formulated products, antioxidant ingredients can be intentionally added to limit oxidation and extending sensory acceptance (10); furthermore, other strategies may be used on meat surface or in packaging structures (11, 12).

Much research has also concluded that chicken livestock conditions greatly influence the final quality of meat and meat products (13). The quick translation from intensive breeding systems to organic ones, which also relies on genetically modified organisms (GMO)-free feeds, no antibiotic use, and high welfare conditions, it is requiring attentive protocols and new indexes to monitor meat quality. In fact, this market section is made of newer products posing diverse stability problems from those known from conventional meats (14).

Besides lipid oxidation, microbial and autolytic enzymatic spoilage are also involved in the qualitative deterioration of the meat during the shelf life (15). Thus, the spoilage processes can cause the production of biogenic amines (BAs) and volatile organic compounds (16, 17).

To check the lipid oxidation, the Thio Barbituric Acid Reactant Species (TBARS) analysis is commonly performed. Oxidation pathways led to high instable products resulting in difficult analyses management (16). TBARS is useful to check the oxidation rate of samples, anyway the information coming from this assay is not so precise and does not inform about the safety (mainly microbiological and chemical spoilage) of the products.

BAs are nitrogenous compounds present in a wide range of food matrices at different percentages in respect of several factors such as the free amino acids profile, the microbiological quality, and the hygienic measures during food processing (18). Generally, meat is considered as an important reservoir of BAs mainly for its content in amino acids from which they are generated (19). The high presence in proteins (amino acids) also depends by the nature of meat, made of muscular tissue where cells are more subjected to BAs influence.

As for all BAs and more in general for bioactive compounds, they are reduced and oxidated creating a complex environment. It is the case of the agmatine that can pass to putrescine and simultaneously be converted to spermine and/or spermidine (20). This suggests that a food rich in amino acids can be already a source of BAs with minor implication of Specific Spoilage Organisms (SSO).

The alteration caused by the microbiological, or oxidation activity, is generally easily identifiable for the color change, the consistency loss, the formation of slime, and the development of off-flavors. All these features alert the consumer leading to refuse the consumption (21). On the contrary, the presence of BAs is more subtle to perceive because most of them are odorless nor do they cause other modifications (14).

In chicken meat, the most prevalent BAs are tyramine, histamine, and polyamines (spermine, SPM; spermidine, SPD; putrescine, PUT cadaverine, CAD). Chicken meat quality is often evaluated by using the SPD/SPM ratio (15); alternatively, other BAs commonly associated with aging and spoilage may be used as quality index. In fact, the sum of some of them can be used as useful index to evaluate the freshness and quality of meat and meat products (22, 23). These biocompounds may indirectly inform about microbial contamination and wrong management, production, and storage conditions of meat and meat products; moreover, since some of them have a toxic effect (vasoactive, psychoactive, or both), their presence can cause serious damage to the consumer, being able to have even lethal effects (24, 25).

Beyond the correct rearing practices and the observance of the best hygiene and manufacturing protocols in the processing, oxidation can be prevented with the use of right packaging according to the storage conditions and the destination of the product (26). About the poultry meat, the polyvinyl chloride (PVC) overwrap (air packaging) is the most common packaging used; this system uses air-permeable and moisture-barrier film to stretch around the meat product. In recent years, an interest on modified atmosphere packaging (MAP) has been observed (27). Vacuum skin packaging (VSP) could also be considered as a type of MAP and is defined as the packaging of a product in a high barrier package from which air is removed to prevent growth of aerobic spoilage organisms, shrinkage, oxidation, and color deterioration. VSP is rarely used for chicken meat because of the less attractive color of the product and the possible leakage occurring in the packaging. Chmiel et al. (9) anyway proved that this is the best method of limiting oxidative phenomena. Gallas et al. (28) refer of the effect of two different MAP compositions on the microbial load and on the BAs accumulation. O_2_ is a promoter of those bacteria which have the enzyme decarboxylase. This peculiarity makes them able to start using SPM and SPD as the main source of Nitrogen (within free amino acids) for producing other BAs. Anyway, this is a low process that is reported to begin after some days of refrigeration. Conversely, concentrations of CO_2_ help in managing the microbial growth prolonging a safe storage. From the same study, it results that using N_2_ does not help nor in limiting BAs accumulation, neither managing the microbial growth. For what concerns TBARs, it can be generally said that O_2_ boosts oxidative phenomena especially when is in combination with other factors as light and heme proteins. Dominguez et al. (29) explain of the contradictory market situation where the presence of O_2_ is negatively correlated with rancidity development while is desirable for the color maintenance.

In the light of these considerations, the present study aimed to use BAI as an unconventional marker of quality and stability for packaged chicken meat, in comparison with traditional index (TBARS, sensory test). The main objective was to find univocal index that makes possible to conjugate food safety needing within sensory attributes. The impossibility of considering TBARS near to BAs values may represent a weak point of this study especially because the two indexes look at different compounds. It is worthy anyway, to start using BAI thoroughly for fresh meat spoilage evaluation. In fact, BAs analysis gives back an image of the general state of conservation of a product also revealing its history.

## Materials and methods

### Origin of the samples and sampling

Samples were provided from an industrial Italian plant (Amadori Group, Cesena, Italy) that breeds and processes chickens meat (antibiotic-free, GMO-free diet, and high welfare/partial free range system meat).

Three cuts of chicken meat were considered: breast filets, drumsticks, and legs (codified B, D, and L, respectively). The cuts were packaged using three types of packaging and conditions: under modified atmosphere or MAP (CO_2_:O_2_-30%:70%); under vacuum or vacuum skin package (VSP); in air-packaging (STRETCH, in which the wrapping consists of a polystyrene tray and a plastic film). A total of 108 samples were analyzed. A specific nomenclature was used for codifying even the packaging. Thus B, D, and L, for breast, drumstick, and leg, were added of M, S, and T, for modified atmosphere, vacuum skin package, and air packaging (stretch), respectively. Samples were stored at +4°C (until to 15 days) in a refrigerated room characterized by reduced thermal abuses and rarely influenced by natural light.

The meat was received directly from the producer already packed under refrigeration. The entire batch was composed of multiple trays (chicken under MAP and STRETCH) and bags (VSP). To allow a correct sampling, new trays and bags were opened for each time of observation. Sampling operations consisted of trays opening, skin removal from drumsticks and legs, meat homogenization through a mechanical blender (Bimby^®^ mixer -Wuppertal, Germany-, mod. TM 31), storage at −40°C in closed falcon tubes, and analysis execution. Four times of observations were taken: immediately after the receiving (T_0_), after 3 (T_3_), 7 (T_7_), and 15 (T_15_) days of refrigerated storage +4°C.

### Physical, physico-chemical, and compositive analyses

Physico-chemical and compositive analyses were carried out on the samples at the beginning of the refrigerated storage time (T_0_), to assess their quality and their optimal managing before starting the experimental plan.

The values of water activity (a_w_) of fresh cuts were obtained with the Aqualab 4 TE kit (Court Pullman, WA, USA). Values of pH were taken with a pH meter (model 3510, Jenway, Stone, UK). All values were measured in triplicate.

Proximate analysis on moisture, proteins, and ashes was obtained following the Association of Official Analytical Chemists procedure (30). Total lipids were measured using a modification of the chloroform to methanol procedure described by Folch et al. (31).

Microelements and vitamins in chicken meat samples have been determined by internal methods, which cannot be detailed for reasons of corporate confidentiality. Selenium and zinc were detected by Inductively Coupled Plasma Mass Spectrometry (ICP-MS) (Agilent 7700 Series and Agilent 7900 Series, Agilent Technologies SPA, Milan, Italy; Thermo IcapQ, ThermoFisher Scientific, Rome, Italy); potassium and phosphorus were determined by Atomic Emission Spectrophotometry (ICP-OES) (Thermo iCap 6300 Radial, ThermoFisher Scientific). Moreover, vitamin PP was detected using LC-(ESI+)-MS/MS system (Agilent 1290 Series; Sciex QTrap 6500+, Sciex, Milan, Italy), while for Vitamins B2 and B12, a system of High Pressure Liquid Chromatography (HPLC) was used (Agilent 1260 Series). Finally, Vitamin B6 was detected by UPLC/MS/MS (Waters Xevo TQS equipped with Aquity H plus, Waters, Milan, Italy).

The composition of the gases inside the packaging was monitored during the storage period using the PBI Dansensor CM9900 instrument (PBI, Padova, Italy).

### Thiobarbituric acid reacting substances assay

A thiobarbituric reactant species test was carried out following the methods of Soyer et al. (32) with some modifications. Raw meat (25 g) was ground in 125 mL of pure water for 2 min to homogenize the mixture. From this, 5 mL were filtered and transferred in falcon tubes (15 mL) with 3 mL of a solution containing trichloroacetic acid (15%, *w*/*v*) and thiobarbituric acid (80 mM) in HCl 0.25 N. Samples underwent a centrifugation step (2,000 rpm for 5 min) to precipitate proteins. After centrifugation, 3 mL were transferred in tapped glass tubes and kept at 40°C for 90 min.

Samples obtained were read at 532 nm with a spectrophotometer UV-VIS (Jenway, Stone, UK) after a further filtration with filters 0.45 μm. All samples were read in double, and data were expressed as mean ± standard deviation.

The calibration curve was obtained using a 1,1,3,3-tetraetoxypropane (Sigma-Aldrich, St. Louis, MO, USA, ≥96%) in methanol, at a concentration range of 0.625–20 μM.

### Biogenic amines' analysis

For this study, putrescine (PUT), cadaverine (CAD), histamine (HIS), tyramine (TYR), spermidine (SPD), and spermine (SPM) were singularly detected. Then, to better comprehend the qualitative state of the meat, a BAI was calculated by summing together PUT, CAD, HIS, and TYR.

The procedure of amines extraction and derivatization was carried out as described by Chaves-Lopez et al. (33) with some modifications.

An aliquot of 4 g of meat was homogenized (in Stomacher Lab blender 400, International PBI, Milan, Italy) with 10 mL of 5% trichloroacetic acid (TCA) (Fluka, Milan, Italia) and centrifuged at 10.000 rpm for 20 min at 4°C (refrigerated centrifuge ALC4237R, ALC International s.r.l.). The supernatant was recovered, and a second extraction was performed as described. The two extracts were put in falcon tubes let to the final volume of 50 mL with 5% TCA acid; the final acid extract was filtered through Whatman 54 paper (Carlo Erba, Milan, Italy).

The derivatization was performed as following. An aliquot of each acid extract (0.25 mL) was mixed with 75 μL of a saturated NaHCO_3_ solution and the pH was adjusted to 11.5 with about 150 μL NaOH. After this, 1 mL of acetone solution containing 10 mg of Dansyl chloride (Fluka) was added to the alkaline amine extract. Derivatized extracts were transferred to a water bath and kept for 60 min at 45°C under agitation (195 stokes) (Dubnoff Bath-BSD/D, International PBI, Milan, Italy). The residual dansyl chloride was removed by adding 100 μL of 300 g/L ammonia solution (Carlo Erba). Extracts were rapidly transferred in dark conditions at room temperature and left for 30 min. Each sample was brought up to add of 2.5 mL acetonitrile (Carlo Erba) and filter through a 0.22 μm PTFE filter (Alltech, Sedriano, Italy).

BAs detection, identification, and quantification were performed by high-performance liquid chromatography (HPLC) using an Agilent 1200 Series (Agilent Technologies, Milan, Italy), on 10 μl of each samples, with gradient elution, acetonitrile (solvent A) and water (solvent B) as follows: 0–1 min 35% B isocratic; 1–5 min, 35–20% B linear; 5–6 min, 20–10% linear B; 6–15 min, 10% B isocratic; 15–18 min, 35% linear B; 18–20 min, and 35% B isocratic.

The separation of the analytes was carried out using a Waters Spherisorb C18 S3ODS-2 column (3 μm particle size, 150 mm × 4.6 mm I.D.), equipped with a Waters Spherisorb S5ODS-2 guard column. Identification and quantification of PUT, CAD, HIS, TYR, SPD, and SPM were performed by comparing retention times and calibration curves of pure standards.

The calibration curves were linear in the range of concentration between 0.5 and 50 mg/L. The lines of regression calculated have been used to compute the amount of the analytes in samples by interpolation, using external standard method.

Limit of detection, precision, and accuracy of the method was assessed. The accuracy of the method was established by setting up recovery tests by samples with known quantities of the investigated BAs. A spiking and recovery procedure was carried out on each meat sample at three BA levels (2–5–10 mg/L), performing five replicates for each concentration level, quantifying, and subtracting the endogenous amine content. Recovery was calculated for histamine (HIS) (83%), tyramine (TYR) (76%), spermidine (SPM) (72%), and spermine (SPD) (70%).

Linearity was assessed by least squares fitting of six independent seven-point calibration curves in the range 0.5–50 mg/L, coming from separately derivatized aliquots.

The limits of detection (LODs) and the limit of quantification (LOQs) were set on poultry meat samples using signal-to-noise ratio (S/N) of 3 and 10, respectively.

Samples for HPLC analysis were stored at −20 ° C in amber glass vials (max 1 week) until HPLC analysis.

### Descriptive sensory analysis

A sensory test was designed to evaluate the sensorial quality (visive, olfactive, and tactile) of the different meat cuts during the period of observation in refrigerated storage (34, 35).

A panel group of 30 people (21 women and 9 men) were asked to rate the color and the odor of all the samples. A scoring scale ranging from 1 to 5 was used (36), where 1 indicates the absence of negative or uncommon qualitative traits, while 5 the maximum of negative attributes presence (discoloration, strong, or uncommon smell). Furthermore, panelists were asked to put in evidence the presence of off-flavors using the symbols –/+. The undetectability is represented by the –, while the presence and the intensity are represented by the +, ++, and +++. This system allows to understand if consumers may perceive unpleasant smells, and which is their intensity. Considering the difficulty in training people with unfamiliar terms to describe meat aging and its degradation, but also to avoid those specific negative terms that may prejudice the scoring, we preferred to use this tool. At any rate, an explanation of off-odors to participants was given to make sure that typical meat flavors may not be mistaken for negative attributes.

Samples were showed to panelists before and after removing packaging. Samples were codified with random numbers to avoid external influences on liking rating of panelists. All sensory tests and training sessions were carried out in the sensory laboratory of the University of Teramo that fulfils the required standards for these analyses according to ISO 8589:2007, (37).

### Statistical analysis

All determinations were done in triplicate, except where differently indicated. Means and relative standard deviations were calculated. Analysis of variance (ANOVA) was performed to test the significance of the effects of the factor variables (cut, packaging, storage time); differences among means were separated by the least significant differences (LSD) test. Statistical analysis of data was performed using XLSTAT software version 2019.1 for Microsoft Excel (Addinsoft, New York, NY, USA). All results were considered statistically significant at *p* < 0.05.

## Results and discussion

### Quality of the samples

**Figure 1:**
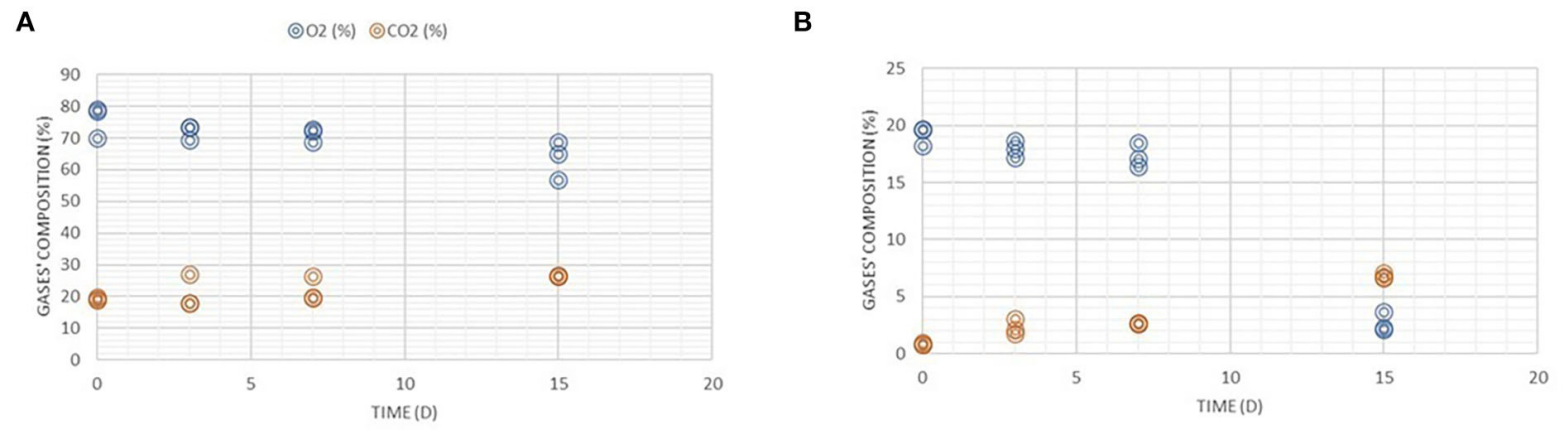
Trend of the gases' composition (%) monitored during the refrigerated storage time (0, 3, 7, and 15 days at 4°C) of all packaged samples in modified atmosphere **(A)** and in air **(B)**.

Chemico-physical parameters (a_w_, 0.966 ± 0.001; pH, 5.78± 0.01), so that the results of compositive analyses (Table 1), of TBARS test (Figure 1) and of BAI index (Figure 7), assessed the high quality and nutritional role of the chicken meat cuts used for the experimental plan. Moreover, Table 2 shows that investigated samples had high nutritional value. Results suggested that all investigated cuts of chicken meat are a good source of selenium, zinc, potassium and phosphorous, and vitamins (B2, B6, B12, PP), which are beneficial to overall body function, and a regular part of a healthy diet. The results are in line with the literature (38).

**Table 1:** Proximate composition (%) of three cuts of chicken meat (without skin).

**Cut**	**Moisture (%)**	**Proteins (%)**	**Lipids (%)**	**Ashes (%)**
Breast	74.97^c^ ± 0.03	21.78^b^ ± 1.56	1.78^a^ ± 0.27	1.39^c^ ± 0.08
Drumstick	70.81^b^ ± 0.05	18.37^a^ ± 0.06	9.45^b^ ± 0.66	1.19^b^ ± 0.01
Leg	69.04^a^ ± 0.05	17.92^a^ ± 0.50	13.00^c^ ± 0.07	1.02^a^ ± 0.01
sign.	***	**	***	**

Data (mean ± standard deviation) followed by different superscript letters, in the same column, are significantly different (LSD test, p < 0.05); asterisks indicate significance at **p < 0.01; ***p < 0.001.

**Table 2:** Minerals and vitamins concentration in cuts of chicken meat investigated.

	**Mineral** ^**§**^ **(mg kg** ^**−1**^ **)**				**Vitamin** ^**§§**^ **(mg kg** ^**−1**^ **)**			
	**Se**	**Zn**	**K**	**P**	**B2**	**B12**	**B6**	**PP**
Breast	0.13 ± 0.02	6.45 ± 0.35	3,795 ± 35.36	2,175.00 ± 7.07	<0.01	<0.001	4.8 ± 0.57	114.5 ± 9.19
Drumstick	0.13 ± 0.01	12.40 ± 1.70	3,170 ± 155.56	1,765.00 ± 49.50	<0.01	<0.001	2.4 ± 0.42	50.6 ± 4.38
Leg	0.14 ± 0.01	19.75 ± 0.78	2,960 ± 325.27	1,650.00 ± 226.27	<0.01	<0.001	2.25 ± 0.21	49.35 ± 1.34

^§^Mineral: Se, Selenium; Zn, Zinc; K, Potassium; P, Phosphorus.

^**§§**^Vitamin: B2, Riboflavin; B12, cobalanin; B6, piridoxine.

### Gas composition trend and role of packaging

**Figure 2:**
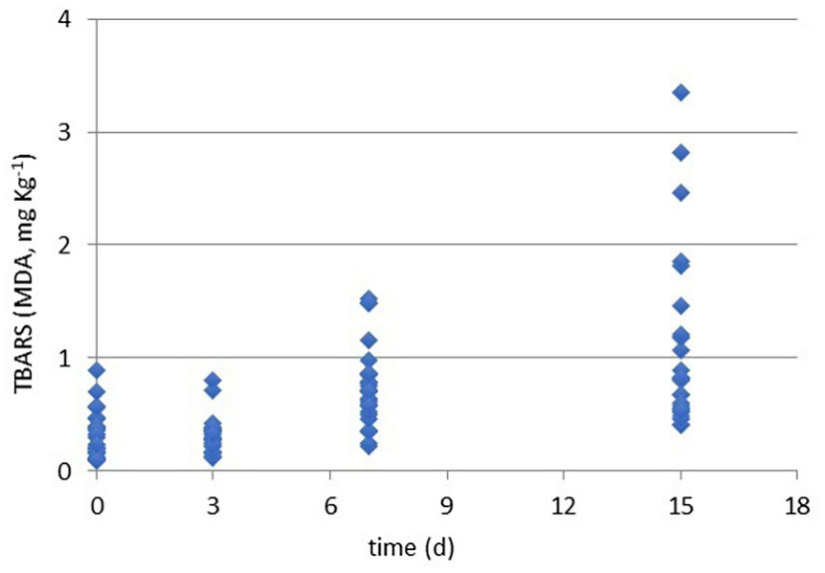
Results of TBARS values (MDA, mg kg^−1^) during the refrigerated storage time (0, 3, 7, and 15 days at 4°C) of all packaged samples.

The composition of gases was monitored for samples in MAP and STRETCH during the refrigerated storage time (Figure 2). For its defined characteristics, vacuum skin pack gaseous composition was not accessed. The use of packaging solutions and specifically of MAP technology protects the food from physical agents also assuring the safety of the products limiting (in combination with cold temperatures) the microbial activity. Like all raw meats, poultry is perishable and subjected to a process of microbial degradation, which can also occur during storage at low temperature; microorganisms of the genus *Pseudomonas* are the major responsible of these phenomena (39).

For MAP and STRETCH, a gradual decrease of O_2_% and increase of CO_2_% were observed, with very relevant differences for stretch at final storage time (T_15_) respect to initial condition (T_0_). CO_2_ in MAP effectively inhibits aerobic deterioration, and the inclusion of O_2_ keeps the color of the meat for a longer period (40). The inclusion of CO_2_ at levels above 20% tends to significantly extend the shelf life of the meat, as aerobic bacteria are well inhibited. Furthermore, the elimination of O_2_ avoids peroxidative phenomena (mainly on lipids, ~10%) if skin is present (41). In any case, the CO_2_ concentration in the package does not exceed 35%, to avoid the collapse of the same. Other deterioration mechanisms to be countered are the aerobic microbial growth and the oxidation of pigments (myoglobin and cytochrome C) more common in skin-free cuts.

VS packaging is a technology that uses the vacuum. So, by removing the air, an adverse environment is created for aerobic pathogens and specific spoilage microorganisms (SSO). In addition, the vacuum-packed product also benefits from the absence of humidity (which is extracted). Therefore, the dry environment prevents the development of some microbiota potentially modifying products' appearance making it, even only visually, healthier from the consumers' point of view (42).

Air permeable wrappings assure protection and food preservation. Moreover, they prevent food from perishing, and extend the shelf-life while maintaining goods' quality attributes. Plastic wrap generally provides protection for food from three aspects: chemical (gases, moisture, and light), biological (microorganisms, insects, and animals), and physical (mechanical damage). Other features as brightness, transparency, and resistance are key attributes of these films. Thanks to the characteristic “memory effect,” the pack maintains the initial tension and appearance, contributing to a perfect presentation of the product. Moreover, it has a perfect permeability to water vapor and O_2_ with a consequent increase in the shelf-life of the product and reduction of weight losses due to dehydration (43).

### TBARS behavior in three chicken cuts differently packaged

As briefly discussed, the control of lipid oxidation is crucial for chicken meat quality. Even if the quantity of lipids is limited, the quality of fatty acids makes this matrix easy for oxidation and peroxidation. Beside the easiness of the analysis, TBARS assay gives limited responses on the reasons of the oxidation' occurrence and does not allow to distinguish lipid to protein oxidation or from other oxidated species (15). Recently Kim et al. (44) have defined the TBARS assay as a method to measure malondialdehyde (MDA), ketones, and oxidation products reporting the TBARS value ≥ of 0.8 mg/kg as perceptible of rancidity in chicken meat.

In the present study, all the samples were screened to evaluate the oxidation rate by the TBARS assay and results are shown in Figure 1. Generally, all the cuts for all packaging solutions produced oxidated species. Moreover, Table 3 reports the ANOVA significance results related to the effects (single and interactions among them) of the considered variables (cut, packaging, and storage time) on TBARS values (MDA, mg kg^−1^).

**Table 3:** Anova matrix results for significative descriptor on TBARS (MA, mg kg-1) and BAI (mg kg^−1^) indices.

**Factor**	**MDA** **(mg kg^−1^)**	**BAI** **(mg kg^−1^)**
Cut	**	**
Packaging	**	**
Storage time (d)	**	**
Cut × Packaging	**	n.s.
Cut × Storage time (d)	**	*
Packaging × Storage time (d)	**	**
Cut × Packaging × Storage time (d)	n.s.	n.s.

Asterisks indicate significance at ^*^p < 0.05, ^**^p < 0.01; n.s. not significant.

**Figure 3:**
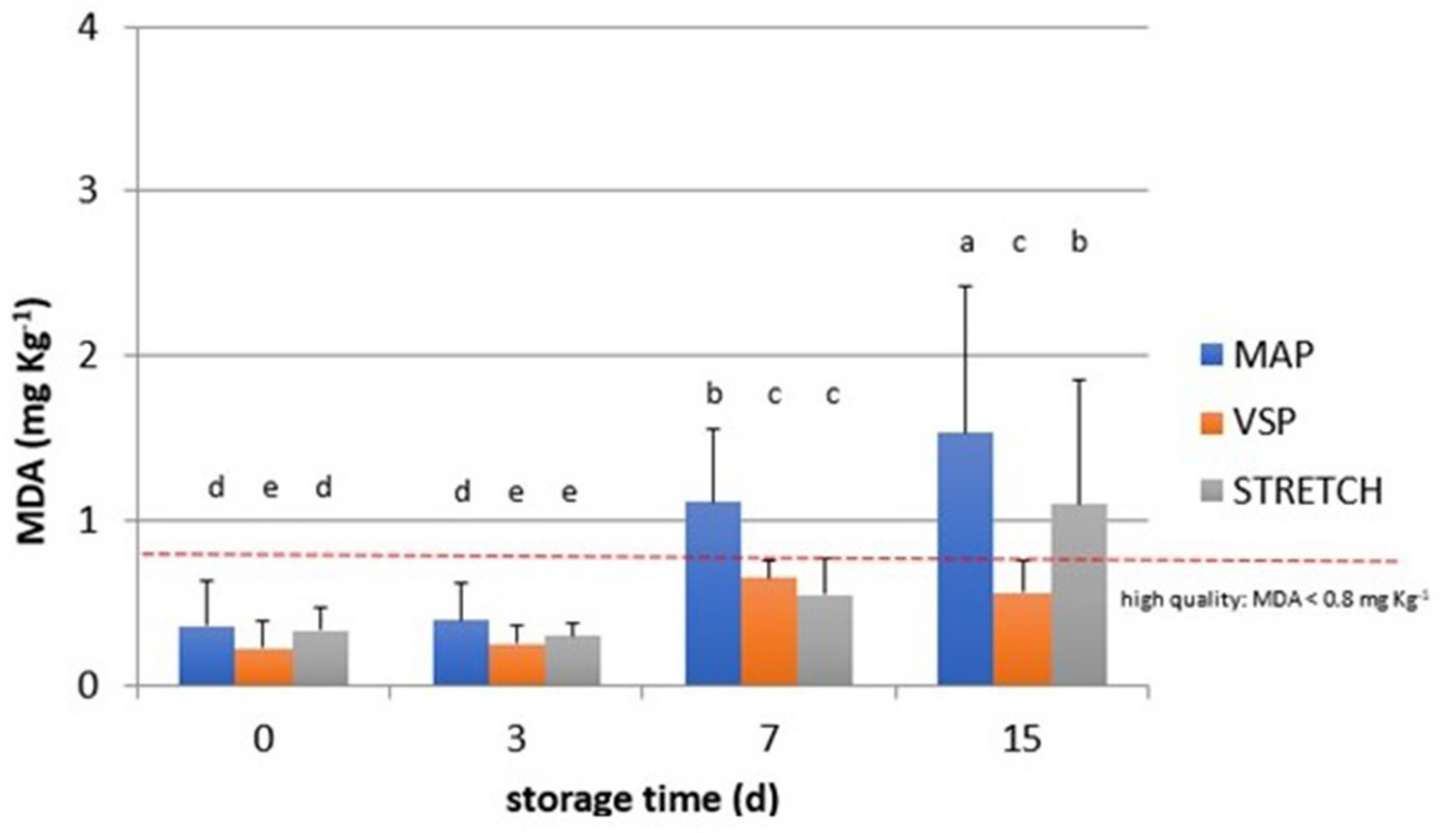
Behavior of TBARS values (MDA, mg kg^−1^) observed in chicken meat packaged in modified atmosphere (MAP), in air (STRETCH), and under vacuum (VSP) and stored at 4°C until 15 days. Data signed with different letters are significantly different (LSD test, *p* < 0.05).

As shown in Figure 3, packaging solutions play an important role in the development of oxidation. In fact, we observed a negative effect of MAP and STRETCH solutions, while vacuum packaging ensures the best protection against the oxidation. As confirmed by other studies (45, 46), the O_2_ concentration in MAP trays can boost the peroxidation especially when packs are sold in display case fridges where temperature fluctuations and light presence interact with O_2_ arising the lipidic oxidation. Samples from this study were stored in a refrigerated room where temperature abuses are reduced, and light presence is limited only when the door is open. Meat industries prefer to not change gas composition of MAP packaging due to the influence on the meat color. Thus, commonly MAP chicken is maintained with 75% O_2_ and 25% CO_2_. This combination ensures the best color appearance limiting the growth of aerobic bacteria as *Pseudomonas spp*. and *Brochotrix thermosphacta* which are typical of meat (47).

**Figure 4:**
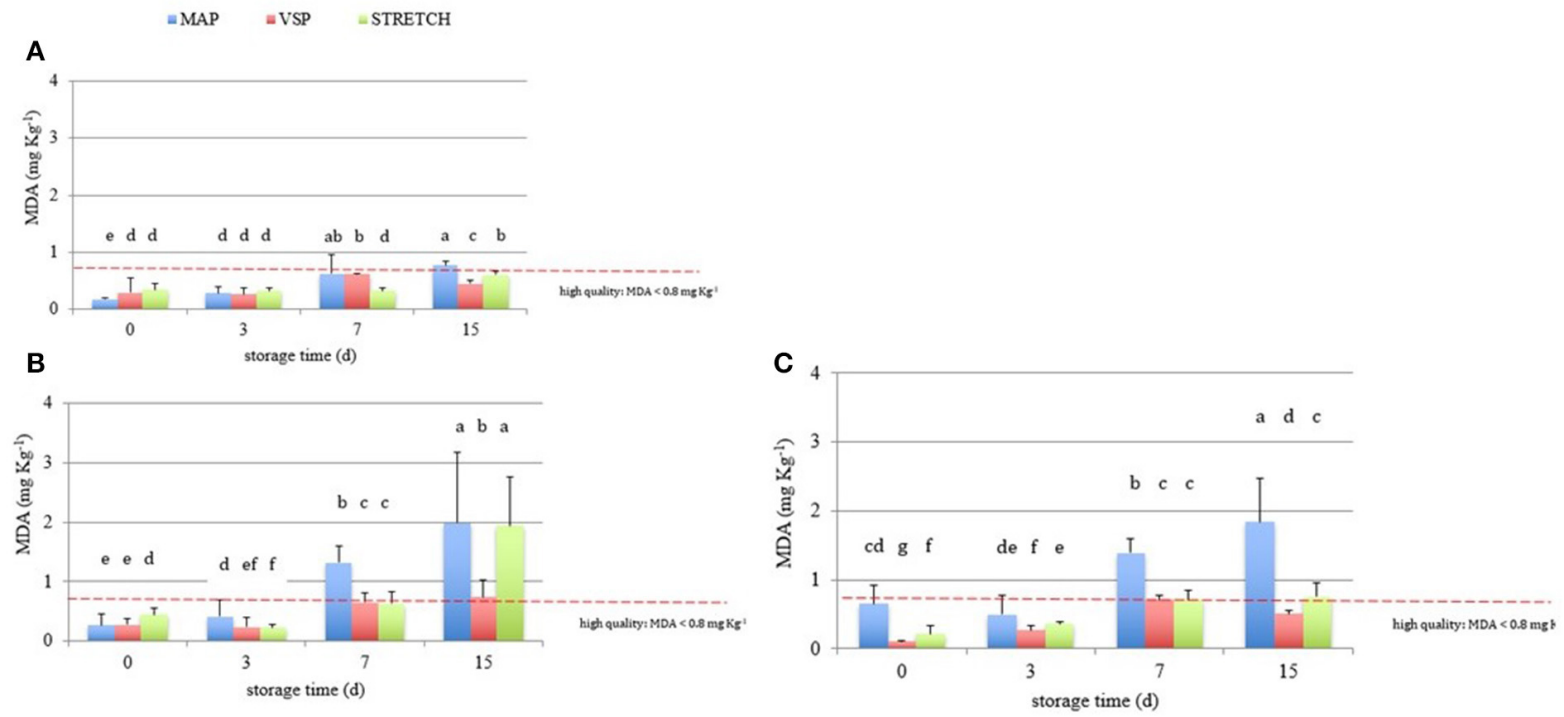
Behavior of TBARS values (MDA, mg kg^−1^) in breast **(A)**, in drumstick **(B)** and in leg **(C)** of chicken meat; data were observed during the refrigerated storage time (0, 3, 7, and 15 days, at 4°C) of samples packaged in modified atmosphere (MAP), in air (STRETCH), and under vacuum (VSP). In each graph, data signed with different letters are significantly different (LSD test, *p* < 0.05).

Data were processed to emphasize the effect of cuts in three different packaging solutions. As shown in Figure 4, breast filets had more stable behavior in respect to TBARS test, independently from the packaging solution. For the other cuts, results were in line with Kim et al. (44). Reasons to explain this trend are mainly conducible to the nature of the muscle, richer in red fibers, with more pH changes due to the lactic acid accumulation during chickens' life (48). Same authors have compared conventional meat with free range and organic one showing that conventional chicken breasts are less prone to oxidation. This characteristic is linked to the chickens' lifestyle and diet. Also, the genetic pathway and the welfare of chickens make important differences on the final oxidation state. Cartoni Mancinelli et al. (49) have demonstrated how slow and medium growing genotypes will develop higher oxidation and volatile compounds (related to oxidation reactions) after cooking in respect of fast growing. Authors linked this trait with the limited movement possibility for fast growing genotype animals who preserve tocols and antioxidants in respect of slow and medium growing genotypes.

Moreover, observing data in Figure 4, at T_0_, all samples are acceptable having lower rancidity. At T_3_, values for breast in all packaging remained similar with an average value of 0.3 MDA eq. What emerges from the comparison among packaging solutions at T_3_ time is that the MAP is the most detrimental for the oxidation rate in all cuts. The close contact with O_2_ can be the main reason to explain this behavior that is particularly observable in drumsticks and legs. By going further along T_7_, this trend is confirmed reaching levels of 1.3 MDA eq. (on average) for the same cuts. In sample BT_7_ (breast air-packed, chilled, and stored for 7 days), TBARs average values are lower than MAP and VSP solutions. Also here, discrepancies of this data can be derived to the closer contact of little amounts of dissolved O_2_ that has got an even stronger impact on the oxidative status. In the case of vacuum packaging solution, the mechanical pressure on food surface increases the drip loss and so the contact with water (50). This is particularly true for those cuts, like breasts, that are not protected by the skin barrier. At any rate, this result makes stronger the idea that TBARs are a screening method generating a picture of the general oxidation rate. By considering valid 0.8 mg/kg as a threshold for rancidity, at T_7_, sensory attributes make all breast samples still acceptable. At the end of the observation (T_15_), the samples confirm the trend with the highest level for MAP packaging for all cuts. The most “inquinated” samples were the drumstick in MAP followed by leg.

### Trend of biogenic amines content

Supplementary Table S1 shows all results concerning biogenic amines content in the investigated samples.

**Figure 5:**
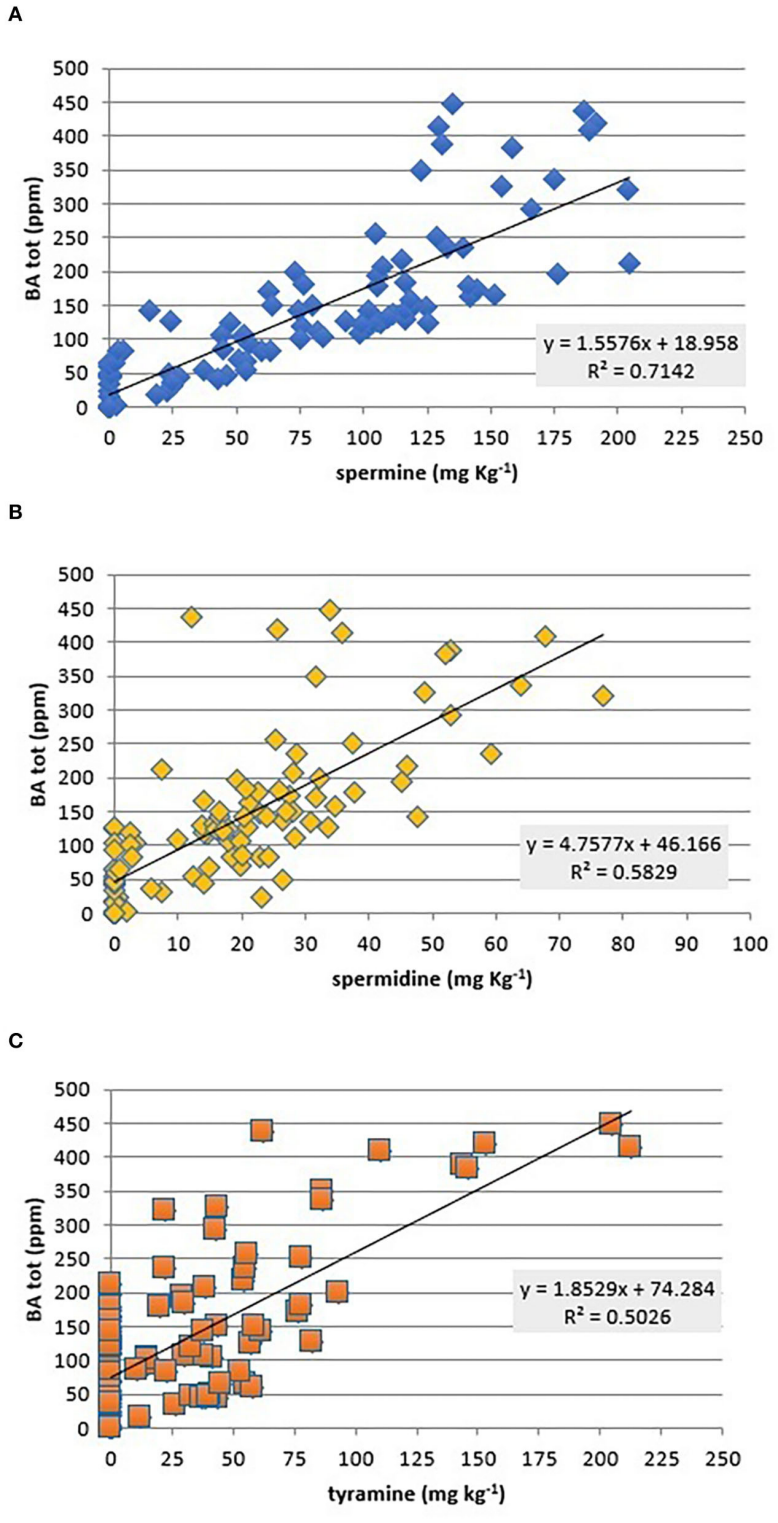
Scatterplot of total biogenic amines (BA, mg kg^−1^) against SPM **(A)**, SPM **(B)**, and TYR **(C)** contents and correlation coefficient values (β) of each single amine (PUT, HIS, and TYR) against total amines found in all investigated chicken meat samples.

Data analysis showed that total BAs are correlated fairly with SPM (β = 0.58), SPD (β = 0.33), and TYR (β = 0.3) (Figure 5), while no significant correlations (*p* < 0.05) were observed with the other amines (Figure 5). As expected in fresh meat, SPM and SPD were the only amines present at a remarkable level, but they seem not harmful to healthy people (51).

Fresh chicken meat ensures a good intake of important BAs like SPM and SPD as highlighted by Bogusławska-Tryk et al. (52). These are independent from fermentative phenomena since they come from proteolytic pathways and the aging of the meat. Not only they cover a functional role during animals' life but also are commonly found in all muscles independently from the shelf life of the meat. For this reason, they are classified as constitutive amines of eukaryotic cells (53); they are also grouped in the polyamine class together with PUT and CAD. Polyamines are small polycations containing two or more amino groups and have been recognized as fundamental for the human health also exerting antioxidant, anti-inflammatory, and an aging protective effect (54).

In respect to SPM and SPD, Silva and Glória (55) have found both in breasts than in tight meat their presence with the major concentration of SPM. These authors were able to trace an increase after 4 days of storage at 4°C while a diminution from the 10^th^ day to the end of the observation (16^th^ day). Our data confirm that SPM is the most abundant BAs in all samples while SPD is less present.

The general trend observed in all cuts for all packaging solutions is a decrease from day 1 to day 3 and an increase from day 7 to day 15. Legs are the most changing cuts with high fluctuations along the time. At the beginning of the storage, values for SPD resulted on average of 20 mg kg^−1^ independently of the packaging (excluding legs packed under vacuum and in MAP where we did not find these amines). Values for polyamines SPD and SPM are generally higher than what reported by Silva and Glória (55) (7 and 18 mg kg^−1^, respectively), Magdy et al. (56), (8 and 56.6 mg kg^−1^, respectively), and Triki et al. (57) (9.78 and 45.03 mg kg^−1^, respectively). Moreover, Magdy et al. (56) did not observe significant differences in SPD and SPM contents among non-vacuum packaging and vacuum packaging in chicken filets. Similarly, Silva and Glória (55) have not registered differences among breast and tight muscles. Our data depict anyway the different behavior of legs in respect of breasts and drumsticks.

Recently, the ration among SPM/SPD has been used to evaluate the freshness of chicken meat (19). As explained, the specificity of these amines makes more reliable its use as a freshness indicator since are not influenced by microorganisms. Anyway, same authors (19) report that high diminutions of these polyamines can indicate an increased microbial activity, since these compounds are used as nitrogen source for bacteria' metabolism. Our data are in line with those of the last cited papers for values of breast filets at T0. Already at T3, we observe a diminution in polyamines possibly imputable to their use as antioxidants (later in the text more details are given), or conversion to other amines.

The major incidence of these amines in samples analyzed can depend by chicken welfare even if few references are available on this. Làzaro and Conte-Junior (58) have anyway demonstrated a higher content of SPM in organic chicken meat with values of 23.67 mg kg^−1^. Chmiel et al. (47), have found values of 18.38 mg kg^−1^ of SPD and 73.41 mg kg^−1^of SPM. Similarly, these authors have not found significant differences among packaging solutions at day 0. In contrast, they have not seen differences on SPM and SPD along the time for packaging solutions while, as aforementioned, our data show a decrease at the beginning of the observation followed by an increase at the end of the storage. This behavior is explained by Ruiz Capillas and Jimenez Colmenero (59), and Balamtsia et al. (60) who observed a steady decrease along the time. The reduction of SPD and SPM can depend by their uptake from bacteria using them (mainly SPM) as nitrogen source (47). The final increase in both amines can be explained as the possible arginine increment during the shelf-life of meat. Triki et al. (57) have seen that poultry (turkey and chicken) is the richest free amino acids containing among meats, with arginine as the main one. The trend registered is an initial availability followed by a steady decrease till the end of the storage where only traces of the amino acid are found. Probably, the extended period of storage here followed can increase proteolytic pathways making arginine available again.

Beyond SPD and SPM functional role that justifies their high presence, some research is explaining the implication in tumoral cells' proliferation (61). These authors have looked for the involvement of polyamines on post-translational activity of proteins with emphasis on SPD being the substrate for the amino acid hypusine; responsible for tumors development and growth. On the other hand, polyamines are recognized for their antioxidant role on cells. Toro-Funes et al. (62) have demonstrated the *in vitro* antioxidant capacity of SPM and SPD at different levels of the peroxidation reaction with good results even when compared to other antioxidants. In consideration of these findings, a major content of polyamines can be explained as an endogenous production of antioxidants in response to a higher demand during chickens' life.

**Figure 6:**
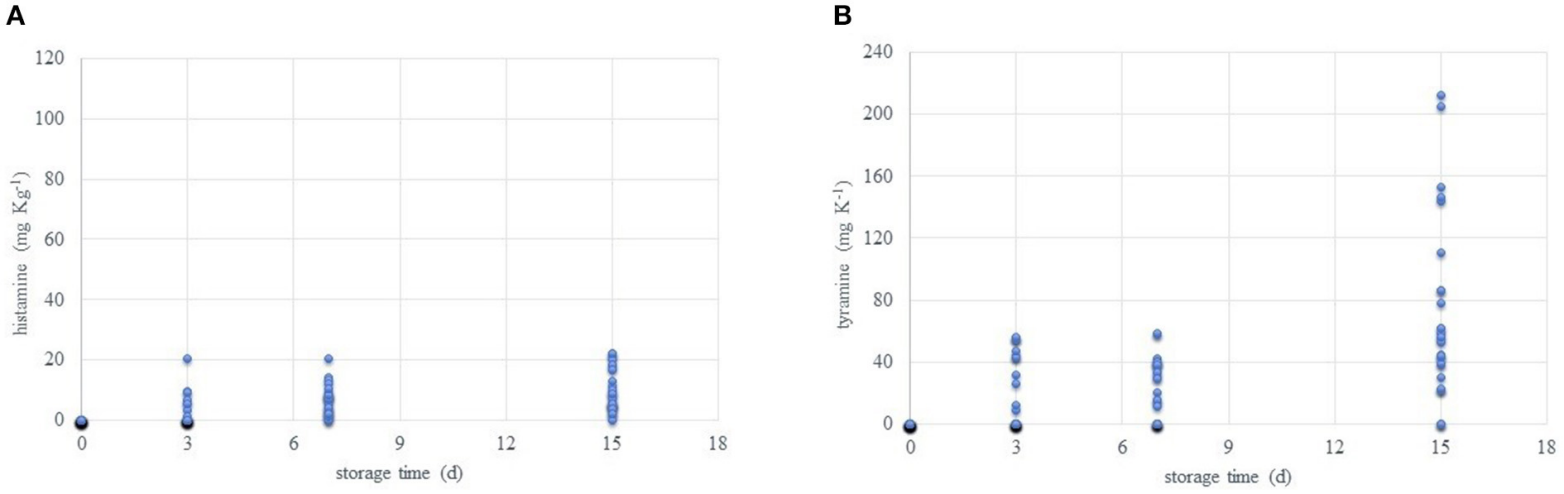
Trends of histamine **(A)** and tyramine **(B)** content (mg kg^−1^) during the refrigerated storage time (0, 3, 7, and 15 days, at 4°C) of all packaged samples.

Supplementary Table S1 reports the values detected for each single amine used for calculating the BAI value during the refrigerated storage period. Figure 6 highlights the growing increase in TYR and HIS along the time. After SPM and SPD, these are the most abundant being also involved in noticed side effects on the health.

Breast meat in air-packaging wrap has got low amounts of HIS and TYR after 3 days of storage. The same happens for drumsticks under vacuum and air-packed. Legs under vacuum register HIS, while TYR resulted in samples packaged under vacuum and in air wrap. Low levels of HIS at early stages of observations can be an index of metabolic activities of tissues since this amine is normally contained in mast cells (63). TYR content is conversely linked to microbial activity mainly imputable to lactic acid bacteria cultures. In particular, the tyramine-producing bacteria are mostly gram positive and belong to the genera *Enterococcus, Lactobacillus, Leuconostoc*, and *Lactococcus* (64). Later, from day 7, all samples present HIS while TYR was not detectable in breast samples. At day 15, both amines increase except for TYR in map breast filets. This low rise is imputable to the bacterial activity, (slowed by the cold temperature) and the continuous availability of free amino acids. Despite this evolution, we cannot consider any of the samples unsafe, capable to disrupt toxicity, or provoking hazards for the human health. This consideration comes from that the accumulation of these amines is detected starting from day 3, and generally fresh chicken meat validity is fixed at 5/6 days if packs are not opened and continuously maintained at +4°C. Moreover, by looking at the recommendations of the Italian Health Ministry, unpacked poultry meat must be consumed in 48 h from the purchase. It must also be considered that the high loss of sensory characteristics along the time would avoid the consumption. Furtherly, no fixed limits from any institution are posed for fresh meat. By guess, if we try to apply current EFSA (65) limits for HIS in fresh fish scombroid and scombroid like species (100–200 mg/kg), we cannot exclude any of the present samples. Of course, the combination of HIS and TYR must be considered more useful for meat and meat products. Later in the next section, more details on indexes' use will be given. Lastly, as recently explained by Sánchez-Pérez et al. (66), BAs may have a synergistic effect on delaying the DAO ability on HIS oxidation. So, considering HIS and TYR and/or other combinations of amines is important because of the well-known negative effects on the human health.

On this, the main symptoms associated with intake of HIS and TYR are nausea, headaches, abdominal cramps, diarrhea, and respiratory disorders (67). Mammalian organisms can degrade the amines, through the mono- and di-aminoxidase (MAO and DAO) enzymes located in the gastrointestinal tract; unfortunately, their effective role is inhibited by a high intake of BAs (66), by alcohol intake and by anti-MAO and anti-DAO drugs (anti-hypertensives, anti-depressants) (68). Some individuals are sensitive to BAs, resulting in symptoms resembling an allergic reaction; HIS poisoning can cause cutaneous, gastrointestinal, neurological (e.g., migraine, burning or itching) or circulatory (hypotension) symptoms (69). In individuals using MAO inhibitor, the ingestion of 60 mg kg^−1^ of dietary TYR can cause migraine, while 100–250 mg kg^−1^ will produce a hypertensive crisis (70). By knowing about these limits, some concerns for sensitive people may arise with certain samples already after 3 days of storage. For example, even if the threshold limit of 60 mg kg^−1^ of TYR is not reached in any case, values near to 50 mg kg^−1^ were registered. This sounds as an alarm, mainly because it is impossible to know how consumers maintain the raw meat. Temperature's fluctuations in home fridges are common to the edge of 7–8°C (71) which, also, when combined with low hygiene maintenance, and microorganisms' presence represents a real hazard. Moreover, consumers may store the meat out of the original packaging unbalancing the BAs' production and reduction equilibrium.

In accordance with (47), the production of PUT is registered after 7 days of storage in chicken filets. In general, PUT and CAD are mainly indices of unwanted microbial activity but in toxic effects are not reported in respect of their isolated activity. However since they inhibit the natural degradation of toxic amines (histamine and tyramine), their presence should still be controlled in meat and meat products and in general meals (27, 66).

In our study, breast filets and legs under air packaging are producing PUT (~2.2 mg kg^−1^) after 7 days of refrigerated storage. Similarly, drumsticks in the same packaging are containing CAD at higher amounts (~6 mg kg^−1^). All cuts in all packaging solutions at T_7_ contain HIS. For breast filets, vacuum pack has got the highest level of this amine, drumsticks exhibit the higher level in MAP packaging while legs have higher HIS in air-packaging. In their study (60), they have not detected HIS nor in air packaging neither in MAP (30% CO_2_, 70% N_2_) until the 8^th^ day of observation. Of course, the different composition of the atmosphere changes the microbial ecology and the kinetic modulating the production of bioactive compounds. Anyway, it is interesting to notice the different influence of cuts origin in respect to the packaging. In general, breast filets are poorer of BAs.

By reviewing, the literature was also possible to see their prevalent incidence on meat. TYR and CAD were found to be the most abundant both in red and white meats (72). In turkey, meat packaged in MAP at different times and with different gaseous compositions, Fraqueza et al. (73) have seen a general trend of BAs increase along the time with particular emphasis on CAD and/or on the sum of PUT, CAD, and TYR.

### Biogenic amines index

The sum of four BAs followed in this study was used as index to better follow and predict meat spoilage and/or quality. BAIs are used for food quality since 1977 when Mietz and Karmas have developed the first one to trace out fresh fish quality. During the years, many researchers have looked for these indexes to monitor food and beverages quality (26, 74, 75). In this study, BAI was assumed including putrescine, cadaverine, histamine, and tyramine. This selection depends on their possible negative effects on the human health and their role on the safety control.

**Figure 7:**
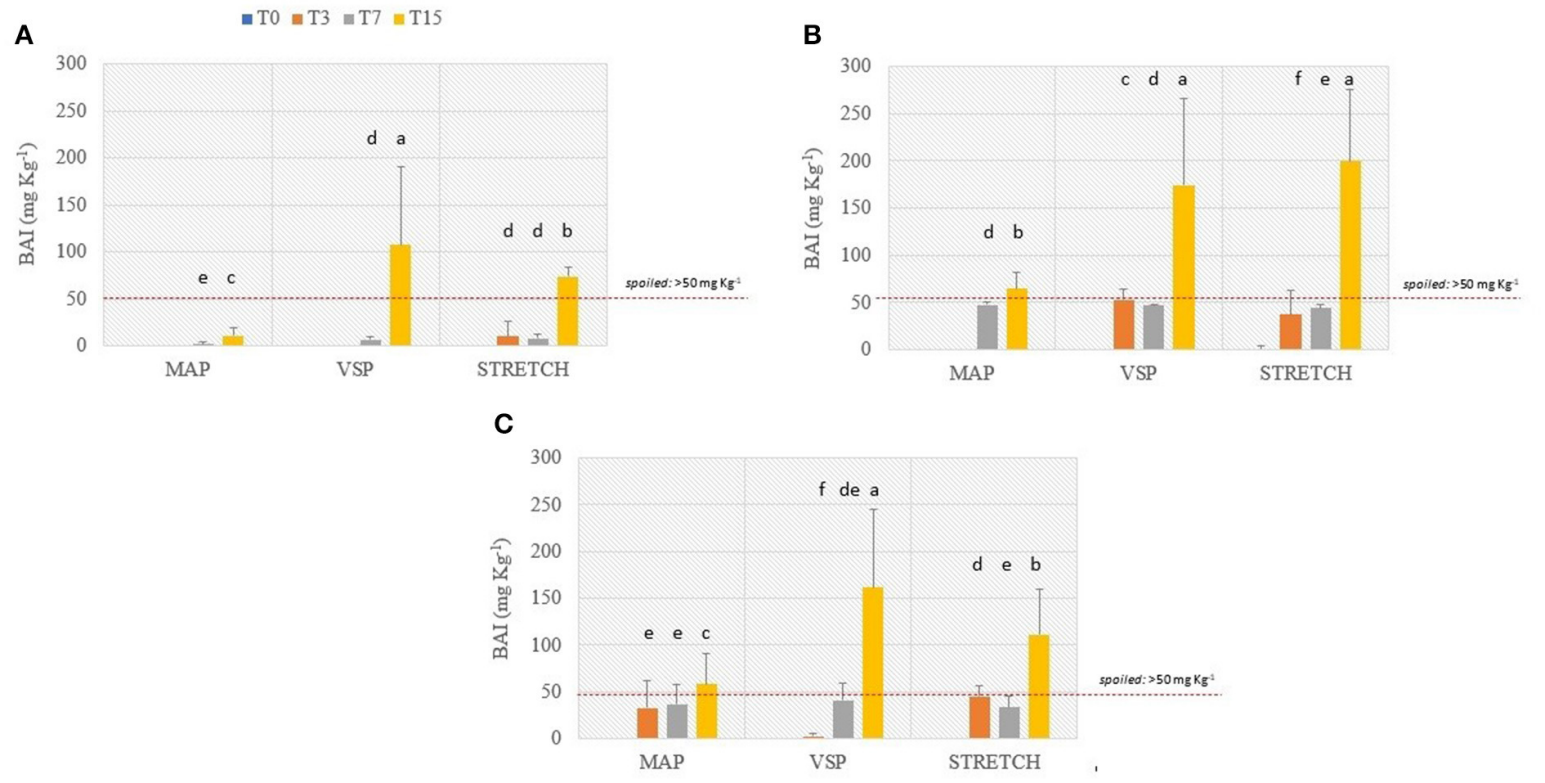
Results of biogenic amines index (BAI, mg kg^−1^) in breast **(A)**, in drumstick **(B)**, and leg **(C)** of chicken meat; data were observed during the refrigerated storage time (0, 3, 7, and 15 days, at 4°C) of samples packaged modified atmosphere (MAP), in air (STRETCH), and under vacuum (VSP). In each graph, data signed with different letters are significantly different (LSD test, *p* < 0.05).

Figure 7 shows the trend of BAI calculated in all investigated samples during the refrigerated storage.

The interest on BAI depends by the fact that single BAs, if contained in a certain amount can be directly toxic (65); moreover, when combined with other factors (alcohol and drugs assumption, and the presence of other BAs), they can exert even increased symptoms of intoxication in both sensitive and less sensitive subjects. Of course, by extending the time of observation, BAs can increase indirectly revealing the microbiological quality of samples. According to the literature (27), when the BAI values are lower than 5 mg kg^−1^, meat is considered of high quality. Then, if BAI values are between 5 and 20 mg kg^−1^, meat is considered acceptable, with the initial spoilage signs; if BAI results between 20 and 50 mg kg^−1^, samples are considered of low quality; finally, BAI values higher than 50 mg kg^−1^ are linked to spoiled meat.

Data from this study reveal a high quality of the raw meat that is highly maintained for at least 7 days of refrigerated storage. Anyway, at T_3_, the BAI index starts to increase in some cases. Graphs in Figures 7A–C are referred to breast filets, drumsticks, and legs, respectively. Considering the limit for the unacceptability fixed at 50 mg kg^−1^, breast filets in all the packaging solutions are slowly accumulating BAs. Samples must be refused after 15 days when packed in VSP and STRETCH. On the other hand, drumsticks are near to the limit already at 7 days of storage in all packaging solutions. The worst solution is VSP where, already after 3 days, BAs content is near to the maximum limit. Legs are very perishable having alarming BAI values at 3 days of storage especially for MAP and STRETCH packaging solutions. For all cuts, graphs illustrate the effectiveness of MAP packaging solution in limiting BAs development in respect to the other two. In many cases, MAP meats are well protected till the end of the shelf-life.

Table 3 shows the ANOVA significance results related to the effects (single and interactions among them) of the considered variables (cut, packaging, and storage time) on BAI (mg kg^−1^). It is possible to observe that each single factor significantly influences the BAI values, as a consequence of what has been discussed above. Our results show that the time is a parameter shaping the final content of BAs. All the reactions allowing BAs accumulation are in fact time-dependent especially when fermentative and/or degradative reactions are conducted under a fixed temperature. The packaging solution too has a direct impact on the final BAI value giving us the impression that the gaseous composition has a role in limiting bacterial activity that is anyway, the first cause of BAs production. This is somehow confirmed by the fact that MAP solutions resulted more protective than others. As seen from the singular BAs trends, breast filets have a different behavior in respect to drumsticks and legs that are more similar. The reasons are above-reported and rely on multiple factors. Probably this intimate difference can explain why the singular effect resulted significative with a p>0.01. At any rate, when cut effect is combined with time or packaging, the significance reduces to *p* > 0.05 or is not significative. This is a good piece of information explaining that raw matter can be comparable for its qualitative attributes globally, and that differences among anatomic parts are not so detrimental in BAI evaluation. Even when looking at two effects values, it is clear that time (storage time) is the most influencing parameter.

A correlation test among TBARS levels (MDA equivalents) and single BAs showed a positive correlation with PUT (β = 0.41), with SPD (β = 0.29), and also with SPM, anyway at very low values (β=0.12). It is known that polyamines are potent antioxidant compounds often synthesized for this specific activity (76). To consolidate this concept, Lázaro et al. (77) have found how polyamines increase after treating chicken breast filets with UV radiations as decontamination method. Although the direct dependency of decarboxylase positive bacteria and BAs levels in food, their presence is not exclusively due to bacterial activity. In the light of these considerations, it can be supposed that TBARS response to oxidation also accounts for oxidated BAs and other compounds related to proteins' oxidation pathways.

### Sensory analyses results

The spoilage processes of meat led to pH changes, appearance changes, slime formation, and can generate secondary metabolism compounds that affect safety and quality of meat products. Changes in volatile fraction of chicken meat samples due to microbial spoilage were correlated with the BAI with high accuracy (78). Those authors proposed a predictive model for BAI using electronic nose, to use it as a tool for routine quality control. This achieves an effective control with low-cost and time saving instruments, as seen for other qualitative parameters (79). From the sniffing test, no sensorial changes were observed on the samples, even when vasoactive amines (TYR and HIS) occurred in chicken meat. Panelists, in fact, attributed low scores for olfactive descriptors testing samples until the 3rd day of storage. Anyway, besides high scores registered at the end of the observation for drumstick and leg packaged in air or under vacuum levels of PUT and CAD detected did not justify the off-flavors spreading (Supplementary Table S1). Even if the aspect is generally considered as the most important factor that affects consumer purchase (21), our results highlight that chicken meat could have a good appearance even when TYR occurs at 30–50 mg kg^−1^ (refer Supplementary Table S1, for drumstick and legs, after 3 days in refrigerated condition). So no correlation can be found between BAs content and sensory. On the other hand, on the same samples, the relationship between sensory data and oxidative reactions resulted different. In our study, sensory analysis demonstrated that when oxidative alterations have occurred, panelists observed significant olfactory and visual changes (Table 4). As affirmed by some authors, oxidative phenomena can generate off-flavors and color changes in chicken meat (80).

**Table 4:** Results of qualitative descriptive analysis (QDA) on three chicken meat cuts during refrigerated storage (+4°C ± 1°C) in three different packaging, for visual and olfactive evaluation.

		**0 days**			**3 days**			**7 days**			**15 days**	
	**Color**	**Odor**	**Off-flavor**	**Color**	**Odor**	**Off-flavor**	**Color**	**Odor**	**Off-flavor**	**Color**	**Odor**	**Off-flavor**
**MAP**												
Breast	1.0 ± 0.0	1.2 ± 0.4 ^a^	-	1.2 ± 0.4	1.0 ± 0.3^a^	-	1.3 ± 0.5^b^	1.9 ± 0.2^a^	+	2.0 ± 0.9^a^	3.2 ± 0.4^a^	+++
Drumstick	1.0 ± 0.0	1.8 ± 0.3^b^	-	1.1 ± 0.2	2.2 ± 0.4^c^	-	1.1 ± 0.2^a^	3.0 ± 0.5^c^	++	1.8 ± 0.5^a^	3.8 ± 0.2^b^	+++
Leg	1.0 ± 0.0	2.0 ± 0.0^b^	-	1.1 ± 0.2	2.1 ± 0.2^c^	-	1.1 ± 0.2^a^	3.2 ± 0.9^c^	++	1.7 ± 0.9^a^	4.0 ± 0.1^c^	+++
**VSP**												
Breast	1.0 ± 0.0	1.0 ± 0.0^a^	-	1.5 ± 0.5	1.2 ± 0.4^b^	-	1.8 ± 0.6^c^	3.0 ± 0.1^c^	++	2.5 ± 0.6^b^	4.5 ± 0.9^cd^	+++
Drumstick	1.0 ± 0.0	2.1 ± 0.2^b^	-	1.1 ± 0.2	2.1 ± 0.8^c^	-	1.5 ± 0.9^c^	3.2 ± 0.8^c^	++	2.8 ± 0.2^b^	5.0 ± 0.0^d^	+++
Leg	1.0 ± 0.0	2.2 ± 0.3^b^	-	1.1 ± 0.2	2.0 ± 0.8^b^	-	1.3 ± 0.5^b^	3.5 ± 0.9^c^	++	2.6 ± 0.5^b^	5.0 ± 0.0^d^	+++
**STRETCH**												
Breast	1.0 ± 0.0	1.0 ± 0.0^a^	-	1.1 ± 0.2	1.3 ± 0.1^b^	-	1.5 ± 0.5^b^	2.5 ± 1.1^b^	+	2.5 ± 0.5^b^	4.0 ± 0.7^c^	+++
Drumstick	1.0 ± 0.0	1.8 ± 0.5^b^	-	1.1 ± 0.2	1.3 ± 0.1^b^	-	1.5 ± 0.3^b^	2.8 ± 0.3^b^	++	3.0 ± 0.8^b^	4.8 ± 0.9^d^	+++
Leg	1.2 ± 0.0	2.2 ± 0.3^b^	-	1.1 ± 0.2	2.3 ± 0.4^c^	-	1.5 ± 0.4^b^	3.0 ± 1.3^c^	++	3.5 ± 0.9^b^	4.2 ± 0.5^c^	+++
	n.s.	***		n.s.	**		**	*		*	**	

Data followed by different superscript letters, in the same column, are significantly different (LSD test, p < 0.05); asterisks indicate significance: at *p < 0.05; at **p < 0.01; at ***p < 0.001; n.s. not significant.

According to the Merriam-Webster dictionary (accessed on March 15, 2022), an off-odor is “an odor that is not natural or up to standard owing to deterioration formation of exudate by bleeding or contamination,” so an extraneous odor that is not conducible to the qualities of the food as known. Commonly, chicken meat has a sweet, not so strong odor with specific notes which are type of this meat. Katiyo et al. (80) have defined six terms for the sensory evaluation of raw chicken legs (fresh chicken, bloody, pungent, fishy, rotten egg, and ammonia-like). The intensity of these flavors may increase and/or decrease defining the quality degradation of the meat. The rise in off-odors more effectively than color indicates the deterioration advancement especially for chicken cuts that notably are less red or generally colored in respect of beef or pork meat.

Texture is the combination of the rheological attributes of a food product perceptible by means of mechanical, tactile, and, where appropriate, visual, and auditory receptors (35); the texture of meat has been widely studied using both sensory evaluation and mechanical methods texture (81). Our study assessed for degree of consistence based on tactile evaluation and highlighted that all samples became softer (scores range: 4.2÷4.8) at the end of storage period, without significant difference among them; in addition, formation of exudate by bleeding was observed already after 3 days of storage for breast packaged under vacuum, causing for these samples a significant higher score for color (Table 4).

## Conclusions

The monitoring of quality decay in chicken meat is not easy and requires constant investigation to assure always optimal products. Reliable indexes based on singular parameters are hard to set up and, when found can somehow mislead the aim limiting the observation to a singular factor such as oxidation for TBARs.

The observation of the samples from the present study demonstrates the direct influence of the packaging on the evolution of reactive species. As expected, vacuum-packed cuts had the lowest oxidation rate if compared with air-packed meat or MAP solutions.

As explained in the article, BAs evolution seems giving higher definition to quality decay monitoring. Anyway, also this parameter talks about the difficulty of choosing an affordable index for qualitative decay of fresh meat. In fact, as shown, packaging solutions assure enough protection when meat is properly produced and stored. Moreover, the absence of the fermentation step limits a lot the BAs occurrence, and probably, although having shown an interesting potential, BAI is more useful in ready-to-eat meat foods, fermented, or suspected products.

Our data assessed that a possible risk can be associated to consume chicken meat; it is also important to highlight that once formed in meat products, BAs are heat stable and will not be destroyed by non-technological treatments (cooking, baking or even canning) (82).

So, to an effective cool chain and MAP packaging, further physical or chemical strategies could be tested to improve the food safety, microbial quality, and shelf life of chicken meat (83). However, BAI can be considered as a useful tool to monitoring the effectiveness of technological treatments, being an indirect index of microbial spoilage and directly involved in toxicity events. Therefore, our results confirmed that while oxidative phenomena cause easily identifiable changes, the occurrence of BAs at potentially toxic levels may be devious for consumer. For this reason, producers of chicken packaged meat should be able to ensure BAs below alarming levels, as well as preventing other alterations due to better known phenomena such as microbiological and oxidative ones.

## Data availability statement

The original contributions presented in the study are included in the article/Supplementary material, further inquiries can be directed to the corresponding author.

## Author contributions

MM, DM, and LE: conceptualization and writing–review and editing. MM and LE: methodology, software, investigation, data curation, and writing–original draft preparation. MM: validation. LE: formal analysis. MM and DM: resources, visualization, and supervision. All authors have read and agreed to the published version of the manuscript.

## Funding

Rural Development Programme for Abruzzo region (PSR ABRUZZO) 2014/2020 Section 16.2. Approved with DPD018/571 of 25 October 2018. CUP C24I19000030009. Project title: Animal welfare of the broiler chicken, and specifically of the chicken raised without the use of antibiotics (FILAVICOLA ABRUZZO). Development of alternative and sustainable breeding and packaging techniques for the production of high-quality food products.

## Conflict of interest

The authors declare that the research was conducted in the absence of any commercial or financial relationships that could be construed as a potential conflict of interest.

## Publisher's note

All claims expressed in this article are solely those of the authors and do not necessarily represent those of their affiliated organizations, or those of the publisher, the editors and the reviewers. Any product that may be evaluated in this article, or claim that may be made by its manufacturer, is not guaranteed or endorsed by the publisher.
