# Corrigendum: Approaching to biogenic amines as quality markers in packaged chicken meat

**DOI:** 10.3389/fnut.2022.1079618

**Published:** 2022-11-09

**Authors:** Luigi Esposito, Dino Mastrocola, Maria Martuscelli

**Affiliations:** Faculty of Bioscience and Technology for Food, Agriculture and Environment, University of Teramo, Teramo, Italy

**Keywords:** biogenic amines, chicken meat, quality, safety, BAI index, TBARS, packaged meat, refrigerated meat

In the published article, there was an error. The ratio of gases composition in MAP packaging was reported inverted.

A correction has been made to **Materials and methods**, *Origin of the samples and sampling*, 2 This sentence previously stated:

“The cuts were packaged using three types of packaging and conditions: under modified atmosphere or MAP (CO_2_:O_2_ - 70%:30%); …”

The corrected sentence appears below:

“The cuts were packaged using three types of packaging and conditions: under modified atmosphere or MAP (CO_2_:O_2_-30%:70%);…”

The authors apologize for this error and state that this does not change the scientific conclusions of the article in any way. The original article has been updated.

## Publisher's note

All claims expressed in this article are solely those of the authors and do not necessarily represent those of their affiliated organizations, or those of the publisher, the editors and the reviewers. Any product that may be evaluated in this article, or claim that may be made by its manufacturer, is not guaranteed or endorsed by the publisher.

