# Supplementary material for: Approaching to biogenic amines as quality markers in packaged chicken meat
Source: Front Nutr. 2022 Sep 1;9:966790. doi: 10.3389/fnut.2022.966790 (PMC9479628; doi:10.3389/fnut.2022.966790)
Supplement: Supplementary file 1 [file Table_1.pdf]

Table S1. Biogenic amines content in all investigated samples (breast, B; drumstick, D; leg, L), in different packaging (in MAP, M; in air, S; under vacuum, T) monitored during refrigerated (+4°C) storage period (at 0, 3, 7 and 15 days)

		PUT	CAD	HIS	TYR	SPD	SPM
T_0							
	BM	n.d.	n.d.	n.d.	n.d.	19.6 ±2.48	111.4 ±8
	BS	n.d.	n.d.	n.d.	n.d.	20.7 ±3.8	106.3 ±5.3
	BT	n.d.	n.d.	n.d.	n.d.	49.2 ± 8.1	122.8 ±7.6
	DM	n.d.	n.d.	n.d.	n.d.	16.9 ± 4.03	101 ±2.5
	DS	n.d.	n.d.	n.d.	n.d.	21 ±2.4	118 ±2.1
	DT	n.d.	n.d.	n.d.	n.d.	16.7 ±2.5	58.4 ±2.1
	LM	n.d.	n.d.	n.d.	n.d	n.d.	44.2 ±1.7
	LS	n.d.	n.d.	n.d.	n.d.	n.d.	53.8 ±3
	LT	n.d.	n.d.	n.d.	n.d.	28.3 ±2.3	82 ±2.8
T_3							
	BM	n.d.	n.d.	n.d.	n.d.	7.4 ±0.08	77.5 ±2.9
	BS	n.d.	n.d.	n.d.	n.d.	20.8 ±2.5	42.9 ±5.8
	BT	n.d.	n.d.	0.7 ±1.5	9,0 ±8.6	n.d.	23.7 ±5.2
	DM	n.d.	n.d.	n.d.	n.d.	12.4 ±1.5	n.d.
	DS	n.d.	n.d.	7.9 ±2.4	47.2 ±6.9	n.d.	n.d.
	DT	n.d.	n.d.	6.2 ±3	31.2 ±1.8	n.d.	n.d.
	LM	n.d.	n.d.	n.d.	49.1 ±8	35.9 ±3.5	95.9 ±9.5
	LS	n.d.	n.d.	3.4 ±0.3	n.d.	21.5 ±0.53	69.8 ±4.6
	LT	n.d.	n.d.	n.d.	45.0 ±0.83	29.9 ±3.4	70.7 ±2.5
T_7							
	BM	n.d.	n.d.	2.7 ±1.9	n.d.	13.9 ±0.15	81.8 ±10.4
	BS	n.d.	n.d.	6.8 ±2.2	n.d.	16 ±0.53	109.7 ±5.2
	BT	2.5 ±1.9	1.1±0.5	4.7 ±0.45	n.d.	18.4 ±3.1	98.1 ±9
	DM	n.d.	n.d.	9.4 ±2.6	37.8 ±4.4	n.d.	17.6 ±0.36

**DS**	n.d.	n.d.	7.4 ±0.5	39.1 ±1.8	n.d.	0.32 ±0.03
**DT**	n.d.	6.22±1.2	2.4 ±0.36	32.8 ±1.4	16.4 ±1.9	33.3 ±2.9
**LM**	n.d.	n.d.	2.7 ±0.9	34.1 ±2.1	13.6 ±1.2	61.2 ±3.8
**LS**	n.d.	n.d.	5.7 ±0.7	35.5 ±1.2	1.6 ±0.15	75.4 ±1.2
**LT**	2.32 ±0.3	n.d.	11.8 ±1.5	20.1 ±6.3	34.2 ±5.2	85 ±0.15

**T_{15}**							
	**BM**	n.d.	n.d.	10.5 ±0.4	n.d.	20 ±2.3	106.4 ±1.8
	**BS**	n.d.	19.2 ±1.6	15.5 ±1.5	183.5 ±1.8	24.9 ±3.9	146.9 ±11.7
	**BT**	n.d.	n.d.	15.4 ±2.6	58.4 ±5.7	13.6 ±1.5	45.2 ±2.3
	**DM**	6.9 ±0.6	2.0 ±0.7	3.4 ±0.2	52.8 ±2.1	29.3 ±5.3	51.3 ±7
	**DS**	9.1 ±1.6	20.4 ±4.6	13.5 ±2.8	131.6 ±4.8	38.9 ±5.5	122.4 ±1.3
	**DT**	70.3 ±2.2	8.9 ±2.1	4.6 ±0.8	115.6 ±2.1	30.2 ±4.5	131 ±8.6
	**LM**	5.9 ±1.4	0.88 ±0.5	8 ±2.4	43.4 ±0.04	66.7 ±3.4	172.5 ±2.8
	**LS**	12.8 ±3.3	56.2 ±4.1	8.4 ±3	83.7 ±1.3	39.0 ±0.57	170.4 ±5.7
	**LT**	42.1 ±1.7	3.0 ±0.5	6.9 ±0.4	58.3 ±4.5	46.9 ±3	115.3 ±2.4
